# Examining the Association between Psychopathic Traits and Fearlessness among Maximum-Security Incarcerated Male Adolescents

**DOI:** 10.3390/children11010065

**Published:** 2024-01-03

**Authors:** J. Michael Maurer, Nathaniel E. Anderson, Corey H. Allen, Kent A. Kiehl

**Affiliations:** 1 The Mind Research Network, 1101 Yale Boulevard NE, Albuquerque, NM 87106, USA; mmaurer@mrn.org (J.M.M.);; 2 Departments of Psychology, Neuroscience and Law, University of New Mexico, Albuquerque, NM 87106, USA

**Keywords:** psychopathy, fearlessness, thrill and adventure seeking, disinhibition, antisocial behavior

## Abstract

Studies have reported positive associations between youth psychopathy scores and measures of ‘fearlessness’. However, prior studies modified fearlessness items to be age appropriate, shifting from assessing hypothetical, extreme forms of physical risk-taking (e.g., flying an airplane) to normative risk-taking (e.g., riding bicycles downhill). We hypothesize that associations between youth psychopathy scores and alternative forms of sensation seeking (i.e., Disinhibition) have been conflated under a false fearlessness label. We tested this hypothesis among incarcerated male adolescents, investigating whether youth psychopathy scores were significantly associated with two different forms of sensation seeking: Disinhibition and Thrill and Adventure Seeking (TAS). Youth psychopathic traits were assessed using the Psychopathy Checklist: Youth Version (PCL:YV), Antisocial Process Screening Device (APSD), Child Psychopathy Scale (CPS), Inventory of Callous and Unemotional Traits (ICU), and Youth Psychopathic Traits Inventory (YPI). Disinhibition and fearlessness (i.e., TAS) were assessed using an unmodified version of the Zuckerman Sensation Seeking Scales (SSS). Consistent with hypotheses, youth psychopathy scores were associated with higher Disinhibition and lower TAS scores. Our results contribute to a growing body of literature suggesting that psychopathic traits, including among adolescents, are not concomitant with physical risk-taking and descriptions of psychopathy including fearlessness distort a precise understanding of psychopathy’s core features.

## 1. Introduction

Individuals meeting criteria for psychopathy are characterized by interpersonal and affective dysfunction, including conning and manipulative behavior, pathological lying, a lack of empathy, guilt, and remorse, and an impulsive, irresponsible lifestyle associated with severe antisocial behavior [1]. Affective or emotional deficits associated with psychopathy include impaired affective empathy [2,3,4], lower emotional intelligence [5,6], and reduced perception of others in pain [7,8]. Furthermore, individuals with elevated psychopathic traits have been characterized by impaired processing of fearful facial expressions [9,10,11,12], poor accommodation of aversive conditioning [13,14,15,16], and reduced fear-potentiated startle [17,18,19]. These processing deficits have led some researchers to describe individuals with elevated psychopathic traits as having a *fearless* temperament. This view was largely popularized by David Lykken’s low fear hypothesis [16,20], suggesting that fearlessness (i.e., a deficiency in the capacity to experience fear) leads to the subsequent development of additional traits and behaviors associated with psychopathy, including risk-taking and engaging in antisocial behavior.

Lykken [20] suggested that fearlessness represents a core feature of psychopathy; however, the evidence to support this notion remains limited (see [21] for an extensive review). Others have argued that rather than being characterized by an inability to subjectively experience fear, individuals with elevated psychopathic traits are more precisely characterized by lower sensitivity to threat-related cues, particularly in complex, goal-oriented contexts [21]. Furthermore, evidence used to support an association between fearlessness and psychopathy, including reduced fear-potentiated startle, has been attributed to attention-related abnormalities [22,23,24]. For example, when directly instructing participants to attend to threat-relevant stimuli, individuals scoring high on psychopathy are characterized by a typical fear-potentiated startle response [23]. Characterizing individuals with elevated psychopathic traits as *fearless* appears to be an imprecise generalization of more specific cognitive abnormalities underlying psychopathy [21,25,26,27,28].

Despite the weak associations previously reported between psychopathy and fearlessness [21,29], some instruments designed to measure psychopathic traits include items assessing fearlessness in generalized ways, including physical risk-taking. For example, the self-report Psychopathic Personality Inventory (PPI-R; [30]) includes an entire subscale comprising items assessing fearlessness. However, other instruments used to measure psychopathic traits do not include items measuring fearlessness, including the Hare Psychopathy Checklist—Revised (PCL-R; [1]), which remains the standard method of assessing psychopathy in clinical and forensic contexts. These conceptual deviations between assessment instruments can contribute to construct drift and inconsistent findings in research [29,31].

The Hare PCL-R was strongly influenced by the clinical criteria established by Hervey Cleckley, who outlined 16 traits characteristic of psychopathy in his influential text, *The Mask of Sanity* [32,33]. Importantly, Cleckley never described individuals scoring high on psychopathy as fearless. In fact, fearlessness is believed to be “counter indicative” of Cleckley’s description of psychopathy ([34], pg. 130). The PPI-R, while incorporating some of Cleckley’s criteria, was also influenced by other etiological theories, namely, Lykken’s low fear hypothesis. Therefore, instruments designed to measure psychopathic traits fundamentally differ with respect to their emphasis on assessing fearlessness, which has important consequences in relation to external measures. For example, our research group previously observed that scores from the Thrill and Adventure Seeking (TAS) subscale from the Zuckerman Sensation Seeking Scales (SSS; [35]), a commonly used measure to assess fearlessness [36,37,38,39], were not significantly correlated with PCL-R scores among incarcerated men and women [29]. Instead, scores from the Psychopathic Personality Inventory—Short Form (PPI-SF; [40]), a modified version of the PPI-R, were associated with higher SSS TAS scores [29]. Disparate findings were observed, as the PPI-SF contains items directly measuring fearlessness (e.g., “I bet it would be fun to pilot a small aircraft alone”), whereas the PCL-R does not. Similarly, studies incorporating the Levenson Self-Report Psychopathy Scale, an instrument containing items largely based on the criteria established by Cleckley (i.e., items that do not directly measure fearlessness), did not observe significant correlations between psychopathic traits and TAS [41,42]. Therefore, certain instruments (i.e., the PPI-SF) continue to suggest that individuals scoring high on psychopathy should be characterized by fearlessness, when limited evidence supports this idea.

Consistent with adults, youth with elevated psychopathic traits have also been characterized by severe affective deficits, including lower emotional intelligence [43], empathy deficits [44,45], reduced processing of others in pain [46], low startle reactivity to aversive stimuli [47,48,49,50,51], and reduced processing of fearful facial expressions [52,53,54,55]. Additionally, consistent with adults, attentional abnormalities contribute to fear-related deficits reported among youth with elevated psychopathic traits. For example, when directly instructing participants to make direct eye contact with individuals expressing fearful facial expressions, youth with elevated psychopathic traits are no longer associated with fear-related processing deficits [56]. Rather than being characterized by fearlessness, Thomson et al. [57] suggest youth scoring high on psychopathic traits are better able to manage fearful situations compared to youth scoring low on psychopathic traits. Specifically, the authors reported that during a virtual reality paradigm, youth scoring high on psychopathic traits responded to fear-induced scenarios with greater sympathetic and parasympathetic activity. These results suggest that youth scoring high on psychopathic traits are able to remain calm and alert when subjectively experiencing fear, giving the appearance as if they were fearless to other individuals.

Unlike adults, instruments developed to assess psychopathic traits among children and adolescents were largely based upon the criteria established by Cleckley and Hare, rather than alternative etiological theories (e.g., Lykken’s low fear hypothesis). Instruments developed to assess youth psychopathic traits include the Hare Psychopathy Checklist: Youth Version (PCL:YV; [58]), Antisocial Process Screening Device (APSD; [59]), Child Psychopathy Scale (CPS; [60]), Inventory of Callous and Unemotional Traits (ICU; [61]), and Youth Psychopathic Traits Inventory (YPI; [62]). Items included within these assessments measure interpersonal dysfunction (e.g., conning and manipulative behavior and pathological lying) and affective deficits (e.g., a lack of empathy, guilt, and remorse). Additionally, both the interview-based PCL:YV and self-report measures (i.e., the APSD, CPS, ICU, and YPI) assess impulsive and irresponsible behavior, whereas the PCL:YV alone measures antisocial/developmental psychopathic traits, including juvenile delinquency and criminal versatility. Therefore, unlike self-report instruments designed to measure psychopathic traits among adults (e.g., the PPI-R), instruments assessing psychopathic traits among children and adolescents do not include items directly measuring fearlessness.

Despite not being included as scoring criteria within these aforementioned instruments, previously published studies have reported significant associations between youth psychopathic traits and fearlessness [50,63,64,65,66,67,68]. Additionally, studies have observed that fearlessness assessed early in life is associated with future callous and unemotional traits [69,70,71]. It should be noted that some instruments and experimental paradigms used to measure fearlessness in youth have shortcomings, which may constrain the generalizability of results obtained. For example, some studies have relied on measures of *perceived* fearlessness, by asking teachers or parents to report how children respond when exposed to stimuli (e.g., toy dragons or scary sounds) or scenarios that may be fear-inducing (e.g., interacting with other children; [69,70,71]), including reporting the number of times children exhibited freezing behavior or sought reassurance from others. Additional studies [50,64,68] have relied upon the teacher-report Child Fearlessness Scale (CFS; [47]), asking teachers about their student’s fearful behavior over the past six months (e.g., “He/she does not seem to be afraid of anything”). However, as outlined by Thomson et al. [57], youth with elevated psychopathic traits often present as if they were fearless, despite subjectively experiencing fear. Therefore, relying on teacher- or parent-report to assess children’s perceived fearlessness may not provide a completely accurate depiction of one’s own perceived level of fearlessness.

In addition to relying on other individuals to assess perceived fearlessness, other studies have directly assessed fearlessness via self-report measures, including the TAS subscale from the Sensation Seeking Scale for Children (SSSC; [72]). TAS items included within the SSSC are believed to be analogous to the SSS TAS subscale used in adults ([67]; pg. 701) and have been associated with higher youth psychopathy scores [63,65,66,67]. Many of the items included within Zuckerman’s SSS scale are not directly applicable to children, and as such, TAS items included within the SSSC were modified for age appropriateness (e.g., “I enjoy the feeling of riding my bike down a big hill” or “I think riding fast on a skateboard is fun”). However, it has been suggested that these age-modified TAS items represent a separate aspect of sensation seeking (i.e., Disinhibition), measuring one’s propensity to engage in exciting experiences and sensations [35], to a greater degree compared to TAS [73,74]. Furthermore, SSSC subscales (e.g., TAS, drug and alcohol attitudes, and social disinhibition) poorly align with Zuckerman’s four sensation seeking domains (e.g., TAS, Disinhibition, Boredom Susceptibility, and Experience Seeking subscales). Due to the different subscales identified using the SSSC and SSS, it is possible that rather than assessing fearlessness (i.e., TAS), previously published studies may have instead reported significant positive associations between youth psychopathic traits and other aspects of sensation seeking, namely, Disinhibition (see [73,74]).

TAS and Disinhibition reflect different aspects of sensation seeking. For example, while TAS reflects *hypothetical* forms of physical risk-taking that individuals plan or desire to engage in, Disinhibition measures *actual* forms of risk-taking that individuals have likely experienced [75,76]. By including age-modified TAS items, previously published studies are no longer measuring hypothetical forms of risk-taking (e.g., flying or jumping out of an airplane), and instead are directly asking participants about forms of physical risk-taking commonly reported among children and adolescents (e.g., riding bicycles or skateboards; [77]). It seems plausible then, that youth scoring high on psychopathic traits were not endorsing SSSC TAS items simply because they did not fear engaging in high-risk sports or physical activity, but rather, they endorsed such items because they represented something that would provide immediate gratification (i.e., having fun engaging in normative childhood and adolescent play activities). Furthermore, Disinhibition scores, rather than TAS scores, have been associated with rule-breaking [78]. Youth with elevated psychopathic traits may have supported items such as riding a bicycle quickly down a hill because their parents and/or guardians previously instructed them not to do so.

Consequently, we believe that previously published studies [63,65,66,67] identified significant associations between youth psychopathy scores and disinhibited, impulsive behavior, masked under a false fearlessness label. In the current study, we investigated the association between youth psychopathic traits (assessed via the PCL:YV, APSD, CPS, ICU, and YPI) and both the SSS Disinhibition and TAS subscales. SSS items were not modified for age appropriateness and, therefore, serve as reliable proxy measures for both disinhibited, impulsive behavior and fearlessness. We hypothesized that youth psychopathy scores (irrespective of the specific instrument used) would be significantly positively associated with SSS Disinhibition scores in both correlation and multiple regression analyses performed. Additionally, we hypothesized that youth psychopathy scores would not be significantly associated with SSS TAS scores, consistent with previous research with adults [29,41,42]. By supporting our hypotheses, this evidence would improve our understanding of youth with elevated psychopathic traits as individuals characterized by a proclivity to engage in disinhibited, impulsive behavior associated with immediate gratification, rather than individuals characterized as incapable of subjectively experiencing fear.

## 2. Method

### 2.1. Participants

Participants included in the current study were incarcerated male adolescents housed at a maximum-security juvenile correctional facility in the state of New Mexico. Participants were recruited as part of the National Institute of Mental Health (NIMH)-funded SouthWest Advanced Neuroimaging Cohort—Youth (SWANC-Y) sample (R01 MH071896; PI: Kiehl). The final sample included incarcerated male adolescents included in this SWANC-Y dataset who had previously completed the SSS (*n* = 220). The majority of these participants completed different instruments assessing youth psychopathic traits, including the PCL:YV (*n* = 217), APSD (*n* = 211), CPS (*n* = 213), ICU (*n* = 216), and YPI (*n* = 216). Participants ranged from 14.13 to 19.81 years of age at the time of SSS data collection (*M* = 17.75 years, *SD* = 1.11 years). Based on National Institute of Health (NIH) race and ethnicity classifications, participants self-identified as American Indian or Alaskan Native (*n* = 26), Black or African American (*n* = 14), Native Hawaiian or other Pacific Islander (*n* = 1), White (*n* = 132), or more than one race (*n* = 8). Additionally, *n* = 39 participants chose not to self-disclose their race. Regarding ethnicity, participants self-identified as Hispanic or Latino (*n* = 163) or not Hispanic or Latino (*n* = 53); four participants chose not to self-disclose their ethnicity. Participants were incarcerated for both violent and non-violent forms of criminal activity that included a range of offenses, including homicide, manslaughter, arson, rape, theft, burglary, drug possession and/or distribution, assault, or fraud.

Initial contact was made with study participants by research staff from the Mind Research Network and informed consent was obtained. Specifically, individuals 18 years of age or older provided written informed consent and individuals younger than 18 years of age provided written informed assent in conjunction with a parent or legal guardian’s informed consent. Interested study participants were excluded from participating in our research study if they were characterized by deficiencies that would impact their ability to properly consent to the overall research study or accurately complete self-report assessments. For example, participants included in the current study were not characterized by a major medical condition (e.g., epilepsy, cancer, severe traumatic brain injury, brain tumor, etc.) and were characterized by a full-scale intelligence quotient (IQ) score above 70 and at least a fourth-grade reading level. Participants received payment consistent with the hourly labor wage of the juvenile correctional facility (roughly USD 1 per hour). All research protocols were approved by the Ethical and Independent Review Services, the Office for Human Research Protections, and the juvenile correctional facility where data collection occurred (Ethic Committee Name: Salus IRB; Approval Date: 8 January 2023 [continuing review]; Approval Code: 15050-09). 

#### 2.1.1. Assessments

PCL:YV: Psychopathic traits were assessed via the Hare Psychopathy Checklist: Youth Version (PCL:YV; [58]), an extension of the PCL-R [1] implemented for use among children and adolescents. The PCL:YV is a 20-item expert-rater assessment, consisting of a semi-structured interview and review of collateral information, including institutional file review. The items included within the PCL:YV are nearly identical to those included within the PCL-R, but are modified for age appropriateness (e.g., “many short-term marital relationships” included in the PCL-R was modified to “unstable interpersonal relationships” in the PCL:YV). Each of the 20 items of the PCL:YV are scored based on the following rating scale: 0 = *does not apply to the individual*, 1 = *applies somewhat to the individual*, or 2 = *definitely applies to the individual*. PCL:YV total scores can potentially range from 0 to 40; the mean PCL:YV total score in the current sample was 24.00 (*SD* = 5.90, range: 2–35, α = 0.82). Consistent with the PCL-R [1,79], the PCL:YV is best characterized by a two-factor and four-facet solution [58,80]. PCL:YV Factor 1 measures interpersonal (Facet 1) and affective (Facet 2) psychopathic traits and PCL:YV Factor 2 consists of lifestyle/behavioral (Facet 3) and antisocial/developmental (Facet 4) psychopathic traits. See Table 1 for full descriptive statistics for all measures included in the current study.

We assessed inter-rater reliability of the PCL:YV using intraclass coefficient correlations (ICC), which were calculated using a two-way, random effects model on average measures, with absolute agreement. Twenty-four participants (10.9% of the sample) had PCL:YV interviews that were double-rated and we observed excellent agreement between raters for the PCL:YV total score (ICC = 0.97, *p* < 0.001). ICCs were not calculated for self-report measures (i.e., the APSD, CPS, ICU, YPI, or SSS), as scores on these assessments were determined via self-report rather than through trained research staff.

APSD: Self-report measures of youth psychopathic traits, including the APSD, CPS, ICU, and YPI, were developed due to the significant time constraints associated with training individuals to score and administer the PCL:YV. Furthermore, collateral information used to score the PCL:YV, including review of institutional files, limits the utility of administering this assessment among non-incarcerated, community youth. The Antisocial Process Screening Device (APSD) can be administered through parent- or teacher-report [59] or individual self-report [81] and consists of 20 items influenced by the criteria established by Cleckley and Hare. Like the PCL-R, each of the 20 items included within the APSD can be scored on a 3-point scale: 0 = *not true at all*, 1 = *sometimes true*, or 2 = *definitely true*. APSD total scores can potentially range from 0 to 40; the mean APSD total score in the current sample was 16.53 (*SD* = 5.14, range: 4–36; α = 0.75). Additionally, factor analysis of the APSD reveals three dimensions [82]: Callous-Unemotional (Factor 1), Impulsivity (Factor 2), and Narcissism (Factor 3) scores. 

CPS: The Child Psychopathy Scale (CPS; [60]) was originally developed using data from the Pittsburgh Youth Study, attempting to measure psychopathic traits in a similar way as the PCL-R. The CPS combined items from the Child Behavior Checklist [83] and the Common Language-Q-Sort [84]. The CPS assesses 13 of the 20 items included within the PCL-R, via a 50-item self-report measure, with binary response options: 0 (*No*) or 1 (*Yes*). CPS total scores can range from 0 to 50; the mean CPS total score in the current sample was 19.67 (*SD* = 6.96, range: 2–42, α = 0.82). Factor analysis of the CPS [85] reveals two subscales: Callous-Unemotional traits (Factor 1) and Antisocial-Impulsive behavior (Factor 2).

ICU: The Inventory of Callous and Unemotional Traits (ICU; [61]) was originally developed to address psychometric limitations associated with the APSD, including a low number of total items devoted towards measuring callous-unemotional (CU) traits. The ICU was developed by expanding upon items included within the APSD CU subscale, resulting in a 24-item self-report measure designed to measure CU traits. Items included within the ICU therefore do not assess impulsive/irresponsible or antisocial/developmental psychopathic traits, instead focusing solely on CU traits. Each of the 24 items included within the ICU are scored on a four-point Likert scale from 0 (*not at all true*) to 3 (*definitely true*). ICU total scores can potentially range from 0 to 72; the mean ICU total score in the current sample was 29.90 (*SD* = 8.64, range: 10–59, α = 0.79). Factor analysis of the ICU reveals three factors [86]: Callousness (Factor 1), Uncaring (Factor 2), and Unemotional (Factor 3) scores.

YPI: The Youth Psychopathic Traits Inventory (YPI; [62]) was largely influenced by Cooke and Michie’s [87] three-factor model of the PCL-R (i.e., assessing interpersonal, affective, and lifestyle/behavioral psychopathic traits, while excluding antisocial/developmental traits). Each of the 50 items included in the YPI are scored on a four-point Likert scale ranging from 1 (*does not apply at all*) to 4 (*applies very well*). YPI total scores can potentially range from 50 to 200; the mean YPI total score in the current sample was 119.09 (*SD* = 20.96, range: 60–184; α = 0.93). Factor analysis of the YPI reveals three factors [62]: Grandiose-Manipulative (Factor 1), Callous-Unemotional (Factor 2), and Impulsive-Irresponsible (Factor 3) scores.

SSS: The Zuckerman Sensation Seeking Scales (SSS) form V [35] is a 40-item self-report measure consisting of binary response options, with one option representing an activity associated with sensation seeking (e.g., “I would like to learn to fly an airplane”) and the other option representing a lack of sensation seeking (e.g., “I would not like to learn to fly an airplane”). Responses corresponding to sensation seeking are given a score of one, whereas the alternative option is given a score of zero. Four factors or subscales are obtained from the SSS, including TAS and Disinhibition, but also Experience Seeking (a desire for experience through the mind and senses, travel, and a non-conforming lifestyle) and Boredom Susceptibility (an aversion to repetition, routine, and dull people). The current study investigated associations between youth psychopathy scores and SSS TAS and Disinhibition scores and, as such, did not investigate other SSS subscales. The mean SSS Disinhibition score in the current sample was 6.70 (*SD* = 2.04, range: 1–10, α = 0.57) whereas the mean TAS score was 6.55 (*SD* = 2.40, range: 1–10, α = 0.71).

KSADS: Additional variables, including rates of substance use disorders and forms of psychopathology, were assessed via trained research staff using the Kiddie Schedule for Affective Disorders and Schizophrenia (KSADS; [88]). Three participants did not complete the KSADS, and as such, it is not known whether they or not they met criteria for forms of psychopathology. The majority of participants in the current sample met criteria for Conduct Disorder (*n* = 217 participants, 95.8% of the sample) and dependence criteria for at least one drug/substance (*n* = 191 participants, 88% of the sample). Drugs/substances assessed via the KSADS included alcohol, cannabis, stimulants, sedatives/hypnotics/anxiolytics, cocaine, opioids, phencyclidine (PCP), hallucinogens, solvents/inhalants, or other (e.g., steroids, ecstasy) substances. On the other hand, study participants were characterized by low rates of attention-deficit/hyperactivity disorder (ADHD; *n* = 15, 6.9% of the sample) and anxiety disorders (e.g., post-traumatic stress disorder, generalized anxiety disorder, obsessive compulsive disorder, etc., *n* = 25, 11.5% of the sample). Forty-one participants (18.9% of the sample) met criteria for an affective disorder (e.g., major depression, dysthymia, depressive disorder not otherwise specified, etc.). No study participants met KSADS criteria for autism spectrum disorders or intellectual disability.

Psychotropic Medication Use: Thirty-three participants (15% of the sample) self-reported using ADHD medications (e.g., Adderall, Ritalin, etc.), 84 participants (38.2% of the sample) reported using anti-depressant medications (e.g., Prozac, Celexa, Zoloft, etc.) and 36 participants (16.4% of the sample) reported using anti-psychotic medication (e.g., Seroquel, Zyprexa, Abilify, etc.). Rates of psychopathology and psychotropic medication use were not included as potential mediating variables in the current study to remain consistent with previously published studies [63,65,66,67].

#### 2.1.2. Data Analysis

We tested our hypotheses by examining whether total scores from the PCL:YV, APSD, CPS, ICU, and YPI were significantly correlated with SSS TAS and Disinhibition scores. Significant effects in the first set of correlations performed reflect those which survived a modified Bonferroni multiple comparison correction (i.e., 0.05/10 or *p* < 0.005). In the second set of analyses, we investigated whether factor scores from each of the youth psychopathy measures (i.e., PCL:YV Factor 1 (interpersonal/affective psychopathic traits) and Factor 2 (lifestyle/behavioral antisocial/developmental psychopathic traits) scores, APSD CU, Impulsivity, and Narcissism scores, CPS CU and Antisocial-Impulsive factor scores, ICU Callousness, Uncaring, and Unemotional scores, or YPI Grandiose-Manipulative, CU, or Impulsive-Irresponsible scores) were significantly correlated with SSS TAS and Disinhibition scores. Significant effects in the second set of correlations performed reflect those which survived a modified Bonferroni multiple comparison correction (i.e., 0.05/26, or *p* < 0.002).

Next, we performed multiple regression analyses, investigating whether subscale scores from each of the five psychopathy instruments were significantly associated with SSS TAS and Disinhibition scores. In these regression analyses, SSS TAS or Disinhibition scores were included as the dependent measure, whereas PCL:YV factor scores, APSD CU, Impulsivity, and Narcissism factor scores, CPS CU and Antisocial-Impulsive factor scores, ICU Callousness, Uncaring, and Unemotional factor scores, or YPI Grandiose-Manipulative, CU, or Impulsive-Irresponsible factor scores were entered as predictor variables in separate analyses performed. In total, ten separate multiple regression analyses were performed (i.e., five with SSS TAS scores entered as the dependent measure and five with SSS Disinhibition scores entered as the dependent measure). Significant effects were those that survived a modified Bonferroni multiple comparison correction (i.e., 0.05/5, or *p* < 0.01).

## 3. Results

### 3.1. Correlation Analyses: Youth Psychopathy Total Scores and SSS Disinhibition and TAS Scores

SSS Disinhibition scores were significantly positively correlated with PCL:YV total scores (*r*[215] = 0.30, *p* < 0.001), APSD total scores (*r*[209] = 0.31, *p* < 0.001), CPS total scores (*r*[211] = 0.37, *p* < 0.001), ICU total scores (*r*[214] = 0.30, *p* < 0.001), and YPI total scores (*r*[214] = 0.29, *p* < 0.001) (see Table 2).

SSS TAS scores were significantly *negatively* correlated with APSD total scores (*r*[209] = −0.24, *p* < 0.001), CPS total scores (*r*[211] = −0.22, *p* = 0.001), and ICU total scores (*r*[214] = −0.28, *p* < 0.001). Additionally, SSS TAS scores were negatively correlated with PCL:YV total scores (*r*[215] = −0.16, *p* = 0.021) and YPI total scores (*r*[214] = −0.17, *p* = 0.012), though these results did not survive multiple comparison correction (see Table 2).

### 3.2. Correlation Analyses: Youth Psychopathy Factor Scores and SSS Disinhibition and TAS Scores

SSS Disinhibition scores were significantly positively correlated with PCL:YV Factor 2 scores (i.e., lifestyle/behavioral and antisocial/developmental psychopathic traits) (*r*[215] = 0.31, *p* < 0.001), APSD CU (*r*[209] = 0.31, *p* < 0.001) and Impulsivity (*r*[209] = 0.25, *p* < 0.001) factor scores, CPS CU (*r*[211] = 0.33, *p* < 0.001) and Antisocial-Impulsive (*r*[211] = 0.32, *p* < 0.001) factor scores, ICU Uncaring (*r*[214] = 0.27, *p* < 0.001) factor scores, and YPI CU (*r*[214] = 0.28, *p* < 0.001) and Impulsive-Irresponsible (*r*[214] = 0.37, *p* < 0.001) factor scores. SSS Disinhibition scores were also positively correlated with PCL:YV Factor 1 (i.e., interpersonal/affective psychopathic traits) (*r*[215] = 0.20, *p* = 0.003) scores and ICU Callousness (*r*[214] = 0.19, *p* = 0.005) and Unemotional (*r*[214] = 0.16, *p* = 0.017) factor scores, though results did not survive multiple comparison correction. APSD Narcissism (*r*[209] = 0.08, *p* = 0.240) scores and YPI Grandiose-Manipulative (*r*[214] = 0.13, *p* = 0.065) factor scores were not significantly correlated with SSS Disinhibition scores (see Table 3).

Additionally, SSS TAS scores were significantly negatively correlated with APSD CU (*r*[209] = −0.26, *p* < 0.001) factor scores, CPS CU (*r*[211] = −0.23, *p* = 0.001) factor scores, ICU Callousness (*r*[214] = −0.25, *p* < 0.001) and Uncaring (*r*[214] = −0.24, *p* < 0.001) factor scores, and YPI CU (*r*[214] = −0.24, *p* < 0.001) factor scores. SSS TAS scores were negatively correlated with PCL:YV Factor 2 (i.e., lifestyle/behavioral and antisocial/developmental psychopathic traits) (*r*[215] = −0.15, *p* = 0.031) scores, APSD Narcissism (*r*[209] = −0.15, *p* = 0.027) factor scores, and CPS Antisocial-Impulsive (*r*[211] = −0.15, *p* = 0.023) factor scores, though results did not survive multiple comparison correction. PCL:YV Factor 1 (i.e., interpersonal and affective psychopathic traits) (*r*[215] = −0.10, *p* = 0.141), APSD Impulsivity (*r*[209] = −0.11, *p* = 0.109) factor scores, ICU Unemotional (*r*[214] = −0.08, *p* = 0.254) factor scores, and YPI Grandiose-Manipulative (*r*[214] = −0.11, *p* = 0.094) and Impulsive-Irresponsible (*r*[214] = −0.09, *p* = 0.202) factor scores were not significantly correlated with SSS TAS scores (see Table 3).

### 3.3. Multiple Regression Analyses: Youth Psychopathy Scores and SSS Disinhibition Scores

SSS Disinhibition scores were entered as the dependent measure and predictor variables included either PCL:YV Factor 1 and 2 scores (Regression #1), APSD CU, Impulsivity, and Narcissism factor scores (Regression #2), CPS CU and Antisocial-Impulsive factor scores (Regression #3), ICU Callousness, Uncaring, and Unemotional factor scores (Regression #4), or YPI Grandiose-Manipulative, CU, or Impulsive-Irresponsible factor scores (Regression #5). Full statistics are reported in Table 4. Here, PCL:YV Factor 2 scores (Regression #1: β = 0.28, *p* < 0.001), APSD Callous-Unemotional (Regression #2: β = 0.27, *p* < 0.001) and Impulsivity factor scores (β = 0.22, *p* = 0.003), CPS Factor 1 (CU scores; Regression #3: β = 0.22, *p* = 0.005) and Factor 2 (Antisocial-Impulsive; β = 0.20, *p* = 0.009) scores, ICU Uncaring factor scores (Regression #4: β = 0.21, *p* = 0.003), and YPI Impulsive-Irresponsible factor scores (Regression #5: β = 0.35, *p* < 0.001) were associated with higher SSS Disinhibition scores. Additionally, YPI CU factor scores (β = 0.17, *p* = 0.043) were associated with higher SSS Disinhibition scores, but did not survive multiple comparison correction. Finally, YPI Grandiose-Manipulative factor scores were moderately associated with reduced SSS Disinhibition scores (β = −0.15, *p* = 0.062). No other significant associations emerged between youth psychopathy scores and SSS Disinhibition scores.

### 3.4. Multiple Regression Analyses: Youth Psychopathy Scores and SSS TAS Scores

SSS TAS scores were the dependent measures and predictor variables remained the same as those included in the first set of analyses performed. Full statistics are reported in Table 5. Briefly, APSD CU factor scores (Regression #2: β = −0.23, *p* < 0.001), ICU Callousness factor scores (Regression #4: β = −0.19, *p* = 0.009), and YPI CU factor scores (Regression #5: β = −0.29, *p* = 0.001) scores were associated with lower SSS TAS scores. Additionally, CPS Factor 1 (CU scores; (β = −0.20, *p* = 0.012) and ICU Uncaring factor scores (β = −0.17, *p* = 0.021) scores were associated with lower TAS scores, but results did not survive multiple comparison correction. Other subscale scores included in these multiple regression analyses were not significantly associated with SSS TAS scores.

## 4. Discussion

The current study investigated the relationship between youth psychopathy scores, assessed via both expert-rater assessments (i.e., the PCL:YV) and self-report measures (i.e., the APSD, CPS, ICU, and YPI), and Disinhibition and fearlessness (i.e., TAS) measured via the SSS among maximum-security incarcerated male adolescents. Both correlation and multiple regression analyses supported our hypotheses, whereby youth psychopathy scores (i.e., both total and subscale/factor scores), regardless of the specific instrument used, were associated with higher SSS Disinhibition scores and lower SSS TAS scores. The lack of a positive association between youth psychopathy scores and TAS scores replicate and extend prior work performed in adults [29,41,42]. Additionally, these results appear at first to contradict studies which have reported significant positive associations between youth psychopathy scores and fearlessness, operationally defined using SSSC TAS scores [63,65,66,67]. However, these prior studies incorporated age-modified TAS items to assess fearlessness, shifting from hypothetical, extreme forms of physical risk-taking (e.g., flying or jumping out of a plane) to normative forms of risk-taking commonly reported among children and adolescents (e.g., riding a bicycle quickly down a hill; [75,76]). It has been suggested that these age-modified TAS items represent Disinhibition, an alternative form of sensation seeking characterized by seeking exciting experiences [35], rather than TAS [73,74]. Therefore, we believe that these previous studies have actually reported associations between youth psychopathy scores and Disinhibition masked under a false fearlessness label. By uniquely associating youth psychopathic traits with Disinhibition, rather than TAS, our results help provide more evidence supporting the idea that individuals scoring high on psychopathy, including adolescents, should not be characterized by fearlessness [21,25,27].

Disinhibition and TAS represent unique forms of sensation seeking that can be further differentiated by impulsivity. For example, Disinhibition is associated with impulsive sensation seeking, whereas TAS is associated with non-impulsive sensation seeking [89,90]. Supporting this position, SSS TAS scores are associated with reduced rates of injuries during bicycle riding [91], skiing [92], and driving [93] and increased use of protective gear, including helmets [94]. This suggests that while individuals scoring high on the TAS engage in physical risk-taking, they are simultaneously acutely aware of the consequences of their actions (i.e., riding safely and wearing protective barriers to reduce injury). This description of TAS is antithetical to our understanding of youth with elevated psychopathic traits, who routinely engage in disinhibited, impulsive behavior [95,96,97], without fully considering the long-term consequences of their actions [58]. For example, youth with elevated psychopathic traits are characterized by higher rates of injuries compared to youth characterized by low levels of psychopathic traits, including traumatic brain injuries [98,99]. Youth with elevated psychopathic traits also engage in activities providing immediate gratification, including substance use (see a recent review by Sakki et al. [100]) and risky sexual behavior [101,102,103], behaviors that are associated with deleterious long-term health consequences.

Our results suggest that youth with elevated psychopathic traits, regardless of the specific instrument used, are more likely to endorse items relating to disinhibited, physically exciting activities (e.g., enjoying parties and engaging in higher rates of substance use and risky sexual behavior). These exciting activities may have been applied to an underspecified fearlessness label [63,65,66,67]. For example, item 25 of the SSS, which is included within the Disinhibition subscale, asks participants whether they like to have new and exciting experiences and sensations, even if a little frightening, unconventional, or illegal [35]. Though not necessarily unconventional or illegal, age-modified TAS items included in these studies may still be considered frightening (e.g., riding bicycles quickly down a hill). Rather than relating to an inability to experience fear when engaging in such normative childhood and adolescent play activities, youth with elevated psychopathic traits may have endorsed such items because they represented exciting experiences associated with immediate gratification (e.g., having fun) or because they were previously instructed not to engage in such activities by parents and/or guardians (e.g., rule-breaking). Disinhibition scores, compared to TAS, have been previously associated with activities that provide immediate gratification and increased instances of rule-breaking [78].

In addition to impulsivity, Disinhibition and TAS relate to criminal behavior and delinquency in opposing directions. For example, Zuckerman [104] suggested that TAS, rather than alternative forms of sensation seeking, was not significantly associated with antisocial behavior. Alternatively, Disinhibition is commonly associated with criminal activity, given the association between this form of sensation seeking and impulsive behavior, including substance use and risky sexual behavior [105,106,107,108]. Zuckerman [104] further distinguished TAS and Disinhibition as “socialized” and “unsocialized” sensation seeking, respectively. Specifically, TAS scores are associated with socially acceptable forms of risk-taking, including mountain climbing and parachute jumping, whereas Disinhibition is associated with socially unacceptable forms of risk-taking, including substance use and impulsive behaviors which increase involvement with the criminal justice system. This differentiation was observed in a study performed by Dåderman et al. [109] who compared SSS scores between juvenile delinquents and air force pilot recruits, observing that juvenile delinquents scored the highest on SSS Disinhibition, whereas air force pilot recruits scored the highest on SSS TAS. Differences between individuals scoring high on the TAS and Disinhibition lies in stark contrast to Lykken [110], who argued that psychopathic murderers and heroic figures including Chuck Yeager, the first man to break the sound barrier via airplane, represented similar individuals, both characterized by fearlessness and a propensity to engage in high-risk, thrill-seeking behavior. While both individuals scoring high on psychopathy and the TAS are characterized by engaging in risky behavior (albeit, different forms of risk-taking), accumulating evidence suggests that these groups of individuals represent fundamentally different groups of people. Beyond fearlessness, individuals scoring high on the TAS are characterized by traits and behaviors that stand in direct opposition to our understanding of youth with elevated psychopathic traits, including considering the long-term consequences associated with their risk-taking behavior [94], lower rates of impulsivity and antisocial behavior [76], and being involved in professions associated with strict rules and guidelines (e.g., air force pilots; [109]). Therefore, given our understanding of individuals scoring high on Disinhibition and TAS, it becomes clearer that youth psychopathy scores should not be positively associated with TAS scores. Studies that have reported such a relationship [63,65,66,67] are therefore likely measuring the association between youth psychopathic traits and alternative measures of sensation seeking, namely, Disinhibition.

Beyond the TAS, other instruments can potentially assess fearlessness, including the behavioral inhibition and activation system (BIS/BAS) scales [111]. Specifically, physical risk-taking involves evaluating the benefits of an action (BAS) and anticipating negative consequences (BIS). Lykken [20] argued that primary psychopaths (i.e., individuals scoring high on psychopathy and low on anxiety) were characterized by an abnormally low BIS and secondary psychopaths (i.e., individuals scoring high on psychopathy and anxiety) were characterized by high BAS. Lykken was largely influenced by Gray’s reinforcement sensitivity theory (RST; [112]), whereby the BIS assesses behaviors that lead to punishment and the BAS measures behaviors that lead to rewards. However, studies suggest that TAS is not only negatively correlated with BIS [113], but also positively correlated with BAS ([113,114]), suggesting that TAS may not be uniquely associated with BIS. As such, while fearlessness can be conceptualized in alternative ways (e.g., BIS), this is precisely the problem with “fearlessness”. While the current study focuses on TAS intentionally, alternative frameworks promote an operationalization of terms that potentially exaggerate the alignment of fearlessness and BIS. The adoption of TAS as an operationalization of fearlessness in the present study is intended to address these misconceptions and highlight the need for more semantic clarity when describing broad features related to individuals scoring high on psychopathy.

### 4.1. Future Directions

In the current study, we did not observe significant positive associations between youth psychopathy total or factor scores and TAS, suggesting that youth with elevated psychopathic traits should not be characterized by undifferentiated fearlessness. This is an important takeaway, considering that recent etiological theories continue to suggest, consistent with Lykken’s [20] low fear hypothesis, that fearlessness is an important component for the future development of psychopathic traits. For example, Frick [115] argues that a fearless temperament gives rise to subsequent psychopathic traits, including a lack of empathy and guilt and conduct problems. Additionally, studies by Waller and colleagues suggest that perceived fearlessness among children measured by parents and teachers is associated with future callous and unemotional traits [69,70,71]. Given the important distinctions clarified in the present work, we would recommend qualifying descriptions like these to clarify that disinhibited, antisocial attitudes (e.g., rule-breaking), impaired aversive learning, and carrying a bias towards immediate, short-term gratification are not well-captured by common descriptions of fearlessness. In fact, terms such as fearlessness are often conflated with other terms, including emotional reactivity, callous-unemotional traits, risk-taking, and excitement seeking [116,117], which may lead researchers to believe that fearlessness represents a core feature associated with youth with elevated psychopathic traits. Rather than being characterized as individuals incapable of experiencing fear, youth with elevated psychopathic traits should instead be characterized by attentional abnormalities that obstruct the salience of emotionally relevant cues [56,118,119] or being able to more effectively manage fearful situations [57].

### 4.2. Limitations

Several limitations of this study should be considered when evaluating the generalizability of our findings. First, we investigated the association between youth psychopathy scores and SSS Disinhibition and TAS scores with a sample of maximum-security incarcerated male adolescents. Compared to women, men score considerably higher on both SSS Disinhibition and TAS [120] and psychopathy assessments which include items relating to fearlessness (e.g., the PPI-R; [121]). Therefore, it is not known whether our results extend to incarcerated female adolescents. Second, our study recruited participants from a maximum-security juvenile correctional facility. Incarcerated youth and youth from the general community significantly differ with respect to a number of variables, most notably, psychopathic traits, history of substance use severity, general intelligence, and trait anxiety [122,123,124]. Future research should investigate the relationship between youth psychopathy scores and SSS TAS and Disinhibition scores among non-incarcerated, community youth to assess generalization to other populations. Additionally, the current study used TAS as a proxy measure of fearlessness, which focuses specifically on physical forms of risk-taking. Not only do individuals scoring high on the TAS endorse forms of physical risk-taking (e.g., parachute jumping, flying an airplane, etc.), such individuals are also characterized by higher rates of non-physical risk-taking, including strategic gambling (e.g., betting on races, sporting events, or games of skill). Future research should investigate whether youth psychopathic traits are associated with a lower propensity to engage in both physical and non-physical forms of risk-taking. Finally, the instrument used to assess sensation seeking and fearlessness in the current study (i.e., the SSS) was developed over 40 years ago and as such, includes outdated language compared to newer approaches. For example, items included within the SSS may not be easily understood by children and adolescents (e.g., “I could conceive of myself seeking pleasures around the world with the jet set”) and incorporate heteronormative assumptions when attempting to assess sensation seeking (e.g., “I would like to meet some persons who are homosexual (men or women)”). Therefore, future studies should consider employing instruments with contemporary language and context.

## 5. Conclusions

In a sample of incarcerated male adolescents, total and factor scores obtained from different measures assessing youth psychopathic traits (i.e., the PCL:YV, APSD, CPS, ICU, and YPI) were associated with higher SSS Disinhibition scores and lower SSS TAS (i.e., fearlessness) scores with both correlation and multiple regression analyses. Our results help improve our understanding of youth with elevated psychopathic traits as individuals who engage in impulsive, reckless, and exciting behavior in order to experience immediate gratification. Additionally, as we did not observe significant positive associations between youth psychopathy scores and TAS, our results add to a growing body of evidence suggesting that it is inappropriate to characterize individuals with elevated psychopathic traits, including youth, with a fearless temperament.

## Figures and Tables

**Table 1:** Descriptive statistics for youth psychopathy scores and SSS Disinhibition and TAS scores.

Variable	Mean	Standard Deviation	Range
PCL:YV Total	24.00	5.90	2–35
PCL:YV Factor 1	6.84	2.96	0–15
PCL:YV Factor 2	14.86	3.13	1–20
APSD Total	16.53	5.14	4–36
APSD Callous-Unemotional	4.73	1.86	0–10
APSD Impulsivity	5.31	1.79	1–10
APSD Narcissism	4.43	2.45	0–13
CPS Total	19.67	6.96	2–42
CPS Callous-Unemotional	10.79	4.23	2–23
CPS Antisocial-Impulsive	8.88	3.68	0–19
ICU Total	29.90	8.64	10–59
ICU Callousness	9.99	4.19	3–27
ICU Uncaring	11.10	4.81	0–22
ICU Unemotional	8.81	2.84	2–15
YPI Total	119.09	20.96	60–184
YPI Grandiose-Manipulative	41.84	10.48	20–68
YPI Callous-Unemotional	36.10	7.07	22–59
YPI Impulsive-Irresponsible	41.16	7.48	15–58
SSS Disinhibition	6.70	2.04	1–10
SSS TAS	6.55	2.40	1–10

Note. PCL:YV refers to the Psychopathy Checklist: Youth Version [58]; PCL:YV Factor 1 refers to interpersonal and affective psychopathic traits and PCL:YV Factor 2 refers to lifestyle/behavioral and antisocial/developmental psychopathic traits; APSD refers to the Antisocial Process Screening Device [59]; CPS refers to the Child Psychopathy Scale [60]; ICU refers to the Inventory for Callous and Unemotional Traits [61]; YPI refers to the Youth Psychopathic Traits Inventory [62]; SSS refers to the Zuckerman Sensation Seeking Scales [35].

**Table 2:** Correlations between youth psychopathy total scores and SSS Disinhibition and TAS scores.

Variable	PCL:YV Total	APSD Total	CPS Total	ICU Total	YPI Total
SSS Disinhibition	*r*[215] = 0.300 * p < 0.001	*r*[209] = 0.306 * p < 0.001	*r*[211] = 0.366 * p < 0.001	*r*[214] = 0.296 * p < 0.001	*r*[214] = 0.290 * p < 0.001
SSS TAS	*r*[215] = −0.156 *p* = 0.021	*r*[209] = −0.241 * p < 0.001	*r*[211] = −0.220 * p = 0.001	*r*[214] = −0.278 * p < 0.001	*r*[214] = −0.170 *p* = 0.012

Note. PCL:YV refers to the Psychopathy Checklist: Youth Version [58]; APSD refers to the Antisocial Process Screening Device [59]; CPS refers to the Child Psychopathy Scale [60]; ICU refers to the Inventory for Callous and Unemotional Traits [61]; YPI refers to the Youth Psychopathic Traits Inventory [62]; SSS refers to the Zuckerman Sensation Seeking Scales [35]. * refers to results which survive a modified Bonferroni multiple comparison correction (0.05/10, or *p* < 0.005).

**Table 3:** Correlations between youth psychopathy factor scores and SSS Disinhibition and TAS scores.

Variable	SSS Disinhibition	SSS TAS
PCL:YV Factor 1	*r*[215] = 0.203, *p* = 0.003	*r*[215] = −0.100, *p* = 0.141
PCL:YV Factor 2	*r*[215] = 0.305 *, *p* < 0.001	*r*[215] = −0.146, *p* = 0.031
APSD Callous-Unemotional	*r*[209] = 0.307 *, *p* < 0.001	*r*[209] = −0.256 *, *p* < 0.001
APSD Impulsivity	*r*[209] = 0.253 *, *p* < 0.001	*r*[209] = −0.111, *p* = 0.109
APSD Narcissism	*r*[209] = 0.081, *p* = 0.240	*r*[209] = −0.152, *p* = 0.027
CPS Callous-Unemotional	*r*[211] = 0.326 *, *p* < 0.001	*r*[211] = −0.228 *, *p* = 0.001
CPS Antisocial-Impulsive	*r*[211] = 0.319 *, *p* < 0.001	*r*[211] = −0.156, *p* = 0.023
ICU Callousness	*r*[214] = 0.191, *p* = 0.005	*r*[214] = −0.248 *, *p* < 0.001
ICU Uncaring	*r*[214] = 0.271 *, *p* < 0.001	*r*[214] = −0.237 *, *p* < 0.001
ICU Unemotional	*r*[214] = 0.162, *p* = 0.017	*r*[214] = −0.078, *p* = 0.254
YPI Grandiose-Manipulative	*r*[214] = 0.126, *p* = 0.065	*r*[214] = −0.114, *p* = 0.094
YPI Callous-Unemotional	*r*[214] = 0.282 *, *p* < 0.001	*r*[214] = −0.242 *, *p* < 0.001
YPI Impulsive-Irresponsible	*r*[214] = 0.371 *, *p* < 0.001	*r*[214] = −0.087, *p* = 0.202

Note. PCL:YV refers to the Psychopathy Checklist: Youth Version [58]; PCL:YV Factor 1 refers to interpersonal and affective psychopathic traits and PCL:YV Factor 2 refers to lifestyle/behavioral and antisocial/developmental psychopathic traits; APSD refers to the Antisocial Process Screening Device [59]; CPS refers to the Child Psychopathy Scale [60]; ICU refers to the Inventory for Callous and Unemotional Traits [61]; YPI refers to the Youth Psychopathic Traits Inventory [62]; SSS refers to the Zuckerman Sensation Seeking Scales [35]. * refers to results which survive a modified Bonferroni multiple comparison correction (0.05/26, or *p* < 0.002).

**Table 4:** Multiple regression analyses with youth psychopathy scores and SSS Disinhibition scores.

Variable	B	SE (B)	β	*t*	Sig.
Regression #1					
PCL:YV Factor 1	0.04	0.05	0.05	0.65	0.517
PCL:YV Factor 2	0.18	0.05	0.28	3.55	<0.001 *
Regression #2					
APSD Callous-Unemotional	0.30	0.08	0.27	3.98	<0.001 *
APSD Impulsivity	0.25	0.08	0.22	2.96	0.003 *
APSD Narcissism	−0.06	0.06	−0.07	−1.01	0.314
Regression #3					
CPS Callous-Unemotional	0.11	0.04	0.22	2.82	0.005 *
CPS Antisocial-Impulsive	0.11	0.04	0.20	2.60	0.009 *
Regression #4					
ICU Callousness	0.05	0.04	0.10	1.35	0.179
ICU Uncaring	0.09	0.03	0.21	2.95	0.003 *
ICU Unemotional	0.07	0.05	0.09	1.37	0.172
Regression #5					
YPI Grandiose-Manipulative	−0.03	0.02	−0.15	−1.87	0.062
YPI Callous-Unemotional	0.05	0.02	0.17	2.03	0.043
YPI Impulsive-Irresponsible	0.10	0.02	0.35	4.43	<0.001 *

Note. Regression #1: R^2^ = 0.095, R = 0.308, *F*(2,216) = 11.192, *p* < 0.001 *; Regression #2: R^2^ = 0.131, R = 0.362, *F*(3,210) = 10.408, *p* < 0.001 *; Regression #3: R^2^ = 0.134, R = 0.366, *F*(2,212) = 16.276, *p* < 0.001 *; Regression #4: R^2^ = 0.091, R = 0.301, *F*(3,215) = 7.054, *p* < 0.001 *; Regression #5: R^2^ = 0.159, R = 0.399, *F*(3,215) = 13.382, *p* < 0.001 *. The dependent measures in these multiple regression analyses were SSS Disinhibition scores. PCL:YV Factor 1 refers to interpersonal and affective psychopathic traits and PCL:YV Factor 2 refers to lifestyle/behavioral and antisocial/developmental psychopathic traits. * refers to results which survive a modified Bonferroni multiple comparison correction (0.05/5, or *p* < 0.01).

**Table 5:** Multiple regression analyses with youth psychopathy scores and SSS TAS scores.

Variable	B	SE (B)	β	*t*	Sig.
Regression #1					
PCL:YV Factor 1	−0.02	0.07	−0.03	−0.35	0.731
PCL:YV Factor 2	−0.10	0.06	−0.13	−1.62	0.108
Regression #2					
APSD Callous-Unemotional	−0.30	0.09	−0.23	−3.33	0.001 *
APSD Impulsivity	−0.02	0.10	−0.01	−0.15	0.882
APSD Narcissism	−0.09	0.07	−0.10	−1.26	0.208
Regression #3					
CPS Callous-Unemotional	−0.11	0.05	−0.20	−2.53	0.012
CPS Antisocial-Impulsive	−0.03	0.05	−0.04	−0.55	0.582
Regression #4					
ICU Callousness	−0.11	0.04	−0.19	−2.61	0.009 *
ICU Uncaring	−0.08	0.04	−0.17	−2.33	0.021
ICU Unemotional	−0.00	0.06	−0.00	−0.06	0.956
Regression #5					
YPI Grandiose-Manipulative	0.00	0.02	0.02	0.18	0.854
YPI Callous-Unemotional	−0.10	0.03	−0.29	−3.30	0.001 *
YPI Impulsive-Irresponsible	0.02	0.03	0.07	0.81	0.417

Note. Regression #1: R^2^ = 0.022, R = 0.148, *F*(2,216) = 2.404, *p* = 0.093; Regression #2: R^2^ = 0.075, R = 0.274, *F*(3,210) = 5.597, *p* = 0.001 *; Regression #3: R^2^ = 0.053, R = 0.231, *F*(2,212) = 5.892, *p* = 0.003 *; Regression #4: R^2^ = 0.086, R = 0.294, *F*(3,215) = 6.663, *p* < 0.001 *; Regression #5: R^2^ = 0.062, R = 0.250, *F*(3,215) = 4.697, *p* = 0.003 *. The dependent measures in these multiple regression analyses were SSS TAS scores. PCL:YV Factor 1 refers to interpersonal and affective psychopathic traits and PCL:YV Factor 2 refers to lifestyle/behavioral and antisocial/developmental psychopathic traits. * refers to results which survive a modified Bonferroni multiple comparison correction (0.05/5, or *p* < 0.01).

## Data Availability

The data presented in this study may be made available upon request from the corresponding author. The data are not publicly available due to the potential for personal identification of participants in the present sensitive population (incarcerated adolescent offenders).
